# Fucoidans as Potential Therapeutics for Age-Related Macular Degeneration—Current Evidence from In Vitro Research

**DOI:** 10.3390/ijms21239272

**Published:** 2020-12-04

**Authors:** Philipp Dörschmann, Alexa Klettner

**Affiliations:** Department of Ophthalmology, Campus Kiel, University Medical Center Schleswig-Holstein UKSH, 24105 Kiel, Germany; philipp.doerschmann@uksh.de

**Keywords:** fucoidan, age-related macular degeneration (AMD), retinal pigment epithelium (RPE), vascular endothelial growth factor (VEGF), *Saccharina latissima*, *Laminaria hyperborea*, oxidative stress, sulfated fucan, brown seaweed

## Abstract

Age-related macular degeneration (AMD) is the major reason for blindness in the industrialized world with limited treatment options. Important pathogenic pathways in AMD include oxidative stress and vascular endothelial growth factor (VEGF) secretion. Due to their bioactivities, fucoidans have recently been suggested as potential therapeutics. This review gives an overview of the recent developments in this field. Recent studies have characterized several fucoidans from different species, with different molecular characteristics and different extraction methods, in regard to their ability to reduce oxidative stress and inhibit VEGF in AMD-relevant in vitro systems. As shown in these studies, fucoidans exhibit a species dependency in their bioactivity. Additionally, molecular properties such as molecular weight and fucose content are important issues. Fucoidans from *Saccharina latissima* and *Laminaria hyperborea* were identified as the most promising candidates for further development. Further research is warranted to establish fucoidans as potential therapeutics for AMD.

## 1. Introduction

### 1.1. Age-Related Macular Degeneration

**Figure 1:**
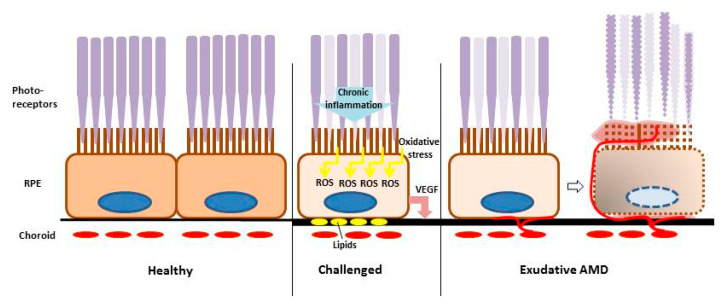
Schematic of (exudative) age-related macular degeneration (AMD) pathogenesis. Retinal pigment epithelium (RPE) cells interact with photoreceptors (purple) and the choroid (red). In AMD development, RPE cells are challenged with oxidative stress, chronic inflammation, and lipid deposits which impair their function and increase VEGF secretion. This may lead to neovascularization from the choroid under and into the retina and to degeneration of the RPE and the photoreceptors; ROS = reactive oxygen species; VEGF = vascular endothelial growth factor.

Age-related macular degeneration (AMD) is the main cause for blindness and severe visual impairment in the industrialized world [1]. It presents in early forms, which are asymptomatic for the patient but detectable for the ophthalmologist, and in two late forms. In the atrophic (“dry”) late form, retinal tissues—most importantly the retinal pigment epithelium (RPE) and secondarily the photoreceptors—degenerate, resulting in a slowly deteriorating vision and finally in large atrophic areas in the macula (geographic atrophy) [2]. In the exudative (“wet”) form of the disease, irregular vessels sprout under and into the retina. The vessels are immature and leaky, causing a fast and severe loss of visual acuity (Figure 1). Additionally, fluid may accumulate in the macula, causing macula edema [3,4]. Only the exudative form of the disease is currently treatable [5]. However, these treatments have to be applied repeatedly, often over several years, and the initial gain in vision is often lost [6]. Currently, no treatments for early forms or the late atrophic form of the disease exist.

AMD is a multifactorial disease, and its pathogenesis is still under investigation. Risk factors are old age, certain genetic polymorphisms, and environmental factors such as smoking or diet [7]. Pathological pathways include lipid dysregulation, inflammation, oxidative stress, and pro-angiogenic signaling [3,8,9] (Figure 1). The latter two will be discussed in more detail.

Oxidative stress is a constant condition of the retina. Due to its function as a light detector, the retina is constantly subjected to (highly energetic) visible light. Furthermore, due to the high oxygen demand of the retina, the oxygen tension surrounding the cells is very high [10]. Photoreceptors and the RPE produce considerable amounts of hydrogen peroxide (H_2_O_2_) in their mitochondria [11]. Furthermore, the concentration of lipids is high because of the abundance of membranes in the photoreceptor’s outer segments (and their fragments, phagocytosed by the RPE). Light waves, especially blue light illumination, can induce reactive oxygen species, and reactive oxygen species can peroxidize lipids and other cellular molecules [11,12,13]. During aging, the RPE accumulates lipofuscin which can act as an additional photosensitizer. The RPE has a high stress tolerance which decreases with age. A high degree of oxidative stress and reduced activation of oxidative stress protection pathways lead to RPE cell death and can induce AMD-like features in animal models [14,15,16,17,18,19].

Oxidative stress is considered a major pathomechanism for all forms of AMD. Pathologic angiogenesis, on the other hand, is indicative of the exudative form of this disease. Here, vessels arise from the choroid and grow beneath or into the retina [4]. These vessels are leaky and can cause edema, and may turn into fibrotic scar tissue [20]. Retinal tissues degenerate rapidly in exudative AMD, which is responsible for the majority of severe loss of visual acuity in AMD [21]. Angiogenesis is a complex procedure involving several angiogenic factors. The major angiogenic factor in health and disease is vascular endothelial growth factor (VEGF) [22]. VEGF is involved in several retinal diseases, such as retinopathy of prematurity, diabetic retinopathy, and age-related macular degeneration [23]. It is secreted by a variety of cells of the retina, but regarding AMD, the RPE is the most important source [2]. VEGF secretion by the RPE is enhanced by many stimuli, such as hypoxia, oxidative stress, and pro-inflammatory stimuli [24,25,26,27]. Enhanced VEGF secretion of the RPE combined with defects in the underlying Bruch’s membrane induce the growth of pathologic vessels from the choroid (choroidal neovascularizations (CNV)) [23,28]. For exudative AMD, anti-VEGF therapy has become the gold standard [5]. However, while vessels regress and some visual acuity may be won under this therapy, generally, long term treatment is needed and visual decline can usually not be prevented in the long term [6].

In addition, VEGF has many physiological functions in the retina [29]. Apart from its tremendous importance in the development of the retinal and choroidal vasculature [30,31], it also exerts functions in the adult retina. VEGF is protective for the RPE and retinal neurons, and needed for supporting endothelial cells and maintaining the structure of the choriocapillaris [32,33,34,35,36]. Current therapies aim to inhibit all VEGF present in the retina [37]. After long-term treatment, atrophic areas are found in many patients [6], and a potential negative effect of long-term inhibition of VEGF on the retina is under debate [38]. A treatment that would be able to reduce (not completely inhibit) VEGF before neovascular angiogenesis occurs would be of great benefit for patients, preventing vessel-associated vision loss and anti-VEGF-associated tissue degeneration. 

### 1.2. Fucoidan

Fucoidans are sulfated polysaccharides (fucans) produced by brown seaweed and echinoderms (such as sea urchins). In brown seaweed, they are part of the cell wall to confer protection against environmental influences. Chemically, they are complex polysaccharides consisting mainly of L-fucose and sulfate ester groups. Fucoidans from brown seaweeds may be branched and may include a variety of additional monosaccharides, resulting in complex and highly variable molecules [39]. Therefore, it is more appropriate to speak of fucoidans in plural, as no single defined fucoidan exists. Fucoidans are of high interest for biomedical applications, as they show a multitude of different bioactivities, e.g., anti-thrombotic, anti-viral, anti-tumor, and many more [39]. Among these bioactivities are also those that render fucoidans interesting for ophthalmological applications, including a potential application in age-related macular degeneration [40]. Bioactivities of major interest regarding AMD are its VEGF inhibition and anti-angiogenic [41,42,43,44,45,46], anti-oxidant [47,48,49,50,51,52], and anti-inflammatory properties [53,54,55,56,57]. However, as mentioned above, fucoidans are highly diverse, and the bioactivity is strongly dependent on the molecular structure of the specific fucoidan, which in turn depends on the species, environmental conditions, and extraction procedures [58,59]. Furthermore, the effects of fucoidans can be converse [60]. For example, fucoidans have been described to be pro-angiogenic as well, mostly depending on the molecular weight of the fucoidan, with low-molecular weight fucoidans generally considered to be pro-angiogenic and high-molecular weight fucoidans considered to be anti-angiogenic [61]. Similarly, fucoidans can act in a pro-inflammatory way, e.g., by activating macrophages [62]. In addition to the molecular properties, the experimental model is also of great relevance for the potential bioactivity tested, as, e.g., the pathway of anti-oxidant protection varies in different cell types [63,64]. Hence, thorough testing in appropriate model systems, regarding aspects such species, molecular properties, and extraction, is of high importance.

### 1.3. Overview of Experimental Models Used in In Vitro AMD Research

#### 1.3.1. In Vitro Models of the RPE

**Figure 2:**
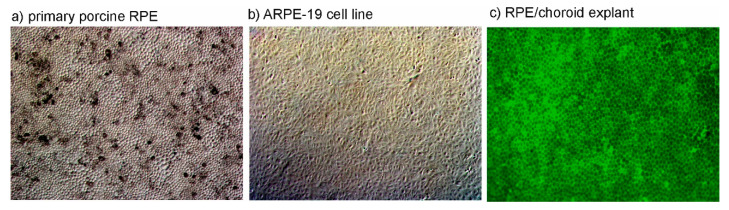
In vitro models to study potential AMD therapeutics: (**a**) primary porcine RPE cells (brighfield, 50×), (**b**) ARPE-19 cells (brightfield, 50×), (**c**) RPE/choroid explants (calcein stain, 50×).

In age-related macular degeneration, the RPE is of high importance in both pathogenesis and treatment [65]. Therefore, suitable models should be applied when testing fucoidans for applications in AMD. The RPE is a highly differentiated cell type with many functions, such as oxidative stress protection, recycling of visual pigment, phagocytosis, cytokine secretion, and many more [66]. For in vitro research, both cell lines and primary cells can be used, and the use of primary cells also requires the choice of suitable species and donor age [67]. A model of great relevance and convenience is the adult RPE cell of porcine origin [68]. While mice are frequently used in experimental research, the murine eye is a less suitable model for the human situation compared to porcine eyes. The porcine eye is of similar shape and size compared to the human eye [69]. Moreover, like man, the pig is a diurnal animal (whilst the mouse is a nocturnal animal). Furthermore, the porcine eye, while not having a macula, possesses a cone-dense visual streak for higher acuity vision [67]. Additionally, the porcine RPE barrier uses similar proteins, as claudin-19 is expressed in the tight junctions of humans and pigs, but not murine RPE [70]. RPE harvested from adult pigs can reach a high degree of differentiation, including morphology, polarization, and barrier properties [68] (Figure 2a). Finally, regarding animal protection, the porcine model is excellent for following the 3R (reduce, replace, refine) principle for the reduction of animal experimentation, as slaughter house waste can be used and no animals need to be sacrificed just for experimentation [71]. 

While primary RPE cells are excellent models, their availability is limited. The use of cell lines can be an acceptable alternative, depending on the objective being studied. The cell line ARPE-19 is a widely used, immortal cell line derived from a 19-year-old donor [72] (Figure 2b). While the original cell line was described to exert several RPE markers, these changed during subsequent sub-passaging [68,72]. However, gene expression patterns concerning phagocytosis, angiogenesis, or apoptosis are comparable to those of sub-cultured human RPE cells [73]. 

Experiments with cultured cells (primary cells or cell line) are valuable models, but do not reflect the complexity of tissue interaction. The RPE in situ is in close contact with the underlying choroid, communicating with this tissue. An elegant way to model this interaction is using RPE/choroid explant models derived from porcine eyes (Figure 2c). For investigation of, e.g., cytokine release, these explants can be cultured in a perfusion chamber, which enables a steady-state equilibrium [74]. In addition to specialized RPE cells, other cell types of ocular origin may be of interest for certain objectives (see below). 

#### 1.3.2. VEGF Secretion 

VEGF is an important factor in pathogenic angiogenesis and the major culprit in exudative AMD. Porcine RPE, both as a primary cell or in explant culture, constitutively secretes considerable amounts of VEGF [75]. ARPE-19 also steadily secretes VEGF, albeit to a lesser degree [76]. While murine VEGF carries a mutation that may interfere with binding properties of conventional VEGF antibodies [77], human and porcine VEGF have high sequence homology and can conveniently be detected in the in cell culture supernatant by commercially available ELISA designed for human studies.

#### 1.3.3. Oxidative Stress Induction

RPE cells are intrinsically highly resistant to oxidative stress [19]. As a rule of the thumb, the more differentiated an RPE cell, the less susceptible it is to oxidative stress [78]. Therefore, oxidative stress protection in the ocular setting can also be investigated in more susceptible cell lines, such as uveal melanoma cell lines [79]. Oxidative stress can be induced by a variety of stressors. Commonly used stressors include hydrogen peroxide (H_2_O_2_) and *tert*-butyl hydroperoxide (TBHP). Importantly, the susceptibility to the respective oxidative stress needs to be established for each individual cell type and cell culture condition [78]. 

## 2. Fucoidans in Ophthalmological Research 

### 2.1. Ophthalmological Studies not Targeting AMD

The interest in fucoidans as potential therapeutics for AMD is rather recent, with a hypothesis paper being published in 2016 by our research group [40]. Some studies exist targeting other ophthalmological diseases, such as diabetic retinopathy, including the effect of the fucoidan from *Fucus vesiculosus* on the RPE cell line ARPE-19. In this study, the fucoidan (100 µg/mL) exerted protective effects on ARPE-19 cells against stress induced by high concentrations of glucose through inhibition of ERK phosphorylation. However, no source of the fucoidan or further characterization was mentioned [63]. Another study investigated the effect of a low-molecular weight fucoidan in a rat model of diabetic retinopathy. The fucoidans were derived from *Laminaria japonica* with a fucose content of 29.5%, a sulfate content of 30.1%, and average molecular weights of 7 and 5.3 kDa. Animals were treated with 50–200 mg/kg/day for four months, while endothelial cells were treated with 12.5–50 µg/mL for up to five days. This fucoidan showed an alleviation of diabetic retinopathy in the animal model and reduced proliferation of endothelial cells in response to VEGF overexpression. VEGF expression in the retina was reduced through an inhibition of the transcription factor HIF-1α [80]. This anti-angiogenic property of the low-molecular weight fucoidan is of particular interest, as generally low weight fucoidans are associated with pro-angiogenic properties [61]. Of note, fucoidans also showed bioactivities that may be protective in diabetes outside the eye, such as the inhibition of dipeptidyl peptidase-IV (DPP-IV), the inhibition of starch hydrolyzing enzymes, and improving insulin-stimulated glucose uptake, thereby protecting the retina by conferring anti-hyperglycemic effects [81,82,83].

Another study addresses the problem of proliferative vitreoretinopathy, a complication of retinal detachment surgery. In this study, a fucoidan from *Fucus vesiculosus* purchased from Sigma Aldrich was used on primary RPE cells and in a rabbit model. This fucoidan (used in concentrations of 50 and 100 µg/mL) inhibited the dedifferentiation of RPE cells in vitro. Furthermore, fucoidan (2000 µg/mL) directly applied into the vitreous of rabbits reduced the development of proliferative vitreoretinopathy in vivo. No further characterization of the fucoidan was described [84]. 

### 2.2. Ophthalmological Studies Targeting AMD

Recently, several studies on fucoidans with regard to their therapeutic potential in regard to AMD have been published. While these publications focus on different aspects, it should be mentioned that so far all these projects were still in vitro, studying ocular cells in culture or RPE/choroidal explants in organ culture. The following sub-chapters will summarize the results of the different studies, regarding VEGF inhibition, oxidative stress protection, and effects on RPE functions. 

#### 2.2.1. VEGF Inhibition and Anti-Angiogenic Properties

The original study identifying fucoidan as a potential therapeutic for AMD focused on VEGF inhibition and anti-angiogenic properties (and effects on the function of RPE cells; see below) was done by Dithmer et al. 2014 [85]. These experiments were conducted with fucoidan from *Fucus vesiculosus* purchased from Sigma Aldrich, used at a concentration of 100 µg/mL. However, no further chemical characterization was conducted on this fucoidan. VEGF-inhibiting properties were tested in three models, the ARPE-19 cell line, primary RPE cells, and RPE/choroid perfusion culture, testing secretion (ARPE-19, perfusion culture) and expression (Western blot—primary RPE; immunofluorescence—primary RPE, ARPE-19). Fucoidan reduced VEGF in all tested parameters, with some variation in the time frames (1–7 d) [85]. In a subsequent study, a fucoidan from *Fucus vesiculosus* purchased from Sigma Aldrich was also tested in primary RPE cells, where it reduced VEGF in the supernatant in a time and concentration-dependent way, being effective at 1–100 µg/mL when tested for 3 d [76]. Furthermore, the anti-angiogenic property of this fucoidan was shown in an angiogenesis assay, when investigating the tube formation of outgrowth endothelial cells, with a significant reduction of tube formation induced by RPE supernatants or VEGF [85]. Of note, the same fucoidan (at the same concentration) did not reduce VEGF secretion in uveal melanoma cells, nor did it exert anti-angiogenic effects here. Conversely, angiogenic effects of the uveal melanoma cell line 92.1 were enhanced by this fucoidan [79]. This finding stresses the dependency of the effect on the cellular context.

Another study tested the effects of defined fucoidans from five species (*Saccharina latissima*, *Laminaria digitata*, *Fucus serratus*, *Fucus vesiculosus*, and *Fucus distichus* subsp. *evanescens* (all from the Baltic Sea, except for *Saccharina latissima* and *Laminaria digitata* (North Atlantic)) and extracted by hot water extraction) on the secretion of VEGF in ARPE-19 and primary RPE cells in concentrations ranging from 1 to 100 µg/mL [76,86]. All tested fucoidans reduced VEGF in the supernatant from ARPE-19 cells after 3 days at almost every concentration tested, with the strongest effect at a concentration of 100 µg/mL. However, in primary RPE cells, only fucoidan from *Saccharina latissima* was effective (10 µg/mL) [76]. Of note, primary RPE cells secreted considerably higher amounts of VEGF per hour than ARPE-19 cells (596.72 vs. 17.35 pg/h). Interestingly, fucoidan from *Saccharina latissima*, while showing the strongest effect, did not exert the strongest VEGF binding affinity [76]. However, it exhibited the highest degree of sulfation, the second highest fucose content, and the highest molecular weight of the five fucoidans [86]. In a study with a crude fucoidan from *Fucus distichus* subsp. *evanescens*, VEGF in the supernatant of ARPE-19 cells was reduced after 24 h (100 and 250 µg/mL), and to lesser degree, after 7 d (250 µg/mL), but not after 3 d [87], indicating that the lower the fucose content, the higher the amounts of uronic acids, and the differences in TPC or molecular weight compared to the purified *Fucus distichus* subsp. *evanescens* fucoidan may have impacts on its ability to reduce VEGF [86].

Additionally, highly pure *Laminaria hyperborea* (North Atlantic) was tested [88,89]. These fucoidans contained 97.0% fucose and a degree of sulfation of 1.7 [90]. The main distinction of these fucoidans was molecular weight, with a high-molecular weight fucoidan (1548 kDa), a medium-molecular weight fucoidan (499 kDa), and a low-molecular weight fucoidan (26.9 kDa) [90]. All three fucoidans reduced the VEGF content in the supernatant of ARPE-19 cells, with the strongest reduction seen in the high-molecular weight fuocidan (50 and 100 µg/mL). Of note, the high and medium-weight fucoidans of *Laminaria hyperborea* also reduced the VEGF content in primary RPE cells (high-molecular weight: 50 µg/mL; medium-molecular weight: 50 and 100 µg/mL) [90]. 

In a different study, fucoidans obtained by enzyme-assisted extraction were studied [91,92]. Tested were different fractions of fucoidan from *Saccharina latissima* (North Atlantic), *Laminaria digitata* (Baltic Sea), and *Fucus distichus* subsp. *evanescens* (Baltic Sea). These fucoidans were of varying purity ranging from 3.9 mol% fucose (*Laminaria digitata*) to 64.7 mol% (*Saccharina latissima*, fraction 2) [91]. Fucoidans from every species reduced VEGF in the supernatant of ARPE-19 cells, with fractions 2 (MW > 800 kDa) and 3 (MW > 800 kDa) of *Saccharina latissima* fucoidan being highly effective at all concentrations tested (1–100 µg/mL), and fucoidans from *Laminaria digitata* (MW 250–450 kDa) and *Fucus distichus* subsp. *evanescens* (MW 200–500 kDa) being less effective [91]. In this study, purity and high molecular weight were associated with better VEGF-inhibiting properties, while an acid-precipitation done in the extraction seemed to lower VEGF-inhibiting function. 

Overall, *Saccharina latissima* and *Laminaria hyperborea* seem to be the most promising species concerning VEGF inhibition in the retinal pigment epithelium. Additionally, efficient VEGF inhibition seems to be dependent on fucose content, molecular weight, extraction procedure, and possibly degree of sulfation. An overview of the fucoidans tested for anti-VEGF bioactivity in the context of AMD is given in Table 1.

#### 2.2.2. Oxidative Stress Protection

The ability to protect against oxidative stress is strongly dependent on the cell used for the experiments. The retina is a highly oxygenic environment, and RPE cells are equipped with responses to counteract oxidative stress, thereby protecting themselves and surrounding cells [19]. 

The fucoidan from *Fucus vesiculosus* from Sigma Aldrich (at a concentration of 100 µg/mL) was able to protect several cell lines of uveal melanoma against cell death induced by H_2_O_2_ [79]. Another study directly compared the effects of fucoidans from five different species regarding oxidative stress protection by testing one uveal melanoma cell line (OMM-1) and the RPE cell line ARPE-19 [76]. These fucoidans have been thoroughly characterized [86]. The fucoidans tested were derived from *Saccharina latissima*, *Laminaria digitata*, *Fucus serratus*, *Fucus vesiculosus*, and *Fucus distichus* subsp. *evanescens* (all from the Baltic Sea except for *Saccharina latissima* and *Laminara digitata* (North Atlantic)) and extracted by hot water extraction. The activities of the fucoidans were tested for concentrations ranging from 1 to 100 µg/mL. All fucoidans protected uveal melanoma cell line OMM-1 from oxidative stress (H_2_O_2_). Conversely, in ARPE-19 cells (challenged with *tert*-butyl hydroperoxide [TBHP]), only *Saccharina latissima* conferred any protection, while fucoidans from *Fucus serratus* or *Fucus distichus* subsp. *evanescens* exacerbated the toxic effect of oxidative stress [76]. These effects are not directly related to molecular weight, as the fucoidans from *Saccharina latissima* and *Fucus vesiculosus* fucoidan had similar molecular weights and only the first one was protective [76,86,93]. Interestingly, the protective effects were not correlated with the radical scavenging ability, as this was higher in fucoidans from *Fucus serratus* (24.6%) and *Fucus distichus* subsp. *evanescens* (10.3%) compared to *Saccharina latissima* (4.5%). As the radical scavenging of fucoidans has been attributed to contaminating phenols [94], this may indicate the induction of protective pathways in ARPE-19 cells, not radical scavenging, as the protective mechanism. This is also supported by a study conducted with crude fucoidan from *Fucus distichus* subsp. *evanescens*. This fucoidan exerted no oxidative stress protection in ARPE-19 cells challenged with TBHP, irrespective of its total polyphenol content [87]. Further research is warranted to elucidate the pathways activated. In addition, the protective effect on ARPE-19 was not associated with the fucose content. While *Saccharina latissima* displayed a high fucose content of 83.8 mol%, fucoidan from *Fucus distichus* subsp. *evanescens* had an even higher fucose content (96.1 mol%). Of note, the tested fucoidan from *Saccharina latissima* had the highest degree of sulfation (0.6), closely followed by *Fucus distichus* subsp. *evanescens* (0.5) [86]. In regard to the tested properties, the anti-oxidative effect on ARPE-19 cells is mainly related to the species from which the fucoidan is derived, which correlates with different structures of the fucoidan tested [89,93]. 

The protective effect of *Laminaria hyperborea* was tested. These fucoidans were highly pure with 97.0% fucose and a degree of sulfation of 1.7. The main difference for these fucoidans was their molecular weight; there was a high-molecular weight fucoidan (1548 KDa), a medium-molecular weight fucoidan (499 KDa), and a low-molecular weight fucoidan (26.9 KDa) [90]. The high-molecular weight fucoidan exerted some protective effect on OMM-1 cells (10 µg/mL), while no protective effect on ARPE-19 cells was observed. 

The protection against oxidative stress was also investigated for fucoidans obtained by enzyme-assisted extraction (*Saccharina latissima* (North Atlantic), *Laminaria digitata* (Baltic Sea), and *Fucus distichus* subsp. *evanescens* (Baltic Sea)). In OMM-1 cells challenged with H_2_O_2_, fucoidans from *Laminaria digitata* showed no protective effect. Conversely, the fucoidan from *Saccharina latissima* (fraction 2) was protective at all concentrations tested, while fucoidans from *Fucus distichus* subsp. *evanescens* showed protection at various concentrations [91]. In ARPE-19 cells challenged with TBHP, a fucoidan from *Laminaria digitata* displayed no protective effect, and fucoidans from *Fucus distichus* subsp. *evanescens* displayed heterogeneous results, with some fractions showing minimal protective effects and others toxic influences. Fucoidans from *Saccharina latissima* displayed only minor effects on cell viability [91]. 

Taken together, these data show that fucoidans from *Saccharina latissima*, extracted by hot water extraction, showed the most promising anti-oxidative effects. An overview of the fucoidans tested for oxidative stress protection in the context of AMD is given in Table 2.

#### 2.2.3. Effects on RPE Survival and Function

Fucoidan from *Fucus vesiculosus* purchased by Sigma Aldrich at a concentration of 100 µg/mL did not exert any toxicity on the ARPE-19 cell line or primary RPE cells tested for up to one week. Similarly, proliferation was not altered by this fucoidan [85]. In another study, the effects of characterized fucoidans from six different algae (*Saccharina latissima*, *Laminaria digitata*, *Fucus serratus*, *Fucus vesiculosus*, *Dictyosiphon foeniculaceus*, and *Fucus distichus* subsp. *evanescens* (all from the Baltic Sea except for *Saccharina latissima* and *Laminaria digitata* (North Atlantic)) and extracted by hot water extraction) [86] on the survival of RPE cell line ARPE-19 and uveal melanoma cell line OMM-1after 24 h of stimulation were tested [95]. The fucoidan from *Fucus serratus* decreased cell viability in a concentration-dependent manner, while the fucoidan from *Laminaria digitata* induced an increase in the viability signal [95]. In OMM-1 cells, viability was also reduced by *Fucus serratus*, and additionally by the fucoidans from *Dictyosiphon foeniculaceus* and *Fucus evanescens.* The fucoidans from *Fucus vesiculosus* and *Laminaria digitata* increased cell viability, and the fucoidan from *Saccharina latissima* produced mixed results [95]. No correlation with any chemical characteristics could be shown, but an effect of purity on cell survival (with less toxicity correlated with better purity) has been discussed by the authors [95]. However, in a study investigating a crude fucoidan extract of *Fucus distichus* subsp. *evanescens*, no toxicity was found for ARPE-19 or primary RPE cells in concentrations up to 250 µg/mL and for up to 7 d of stimulation [87]. 

In another study, the effects on viability of three different fucoidans from *Laminaria hyperborea* were tested. These fucoidans were highly pure with 97.0% fucose and a degree of sulfation of 1.7. The main difference among these fucoidans was the molecular weight, with a high-molecular weight fucoidan (1548 kDa), a medium-molecular weight fucoidan (499 kDa), and a low-molecular weight fucoidan (26.9 kDa) [90]. When applied to the uveal melanoma cell line OMM-1, fucoidans of medium and high-molecular weight reduced the cell viability of these cells (50 and 100 µg/mL), while no effect was seen regarding the viability of ARPE-19 or primary human RPE cells after 24 h. In the study with fucoidans obtained by enzyme assisted extraction (*Saccharina latissima* (North Atlantic), *Laminaria digitata* (Baltic Sea), and *Fucus distichus* subsp. *evanescens* (Baltic Sea)), none of the tested fucoidans exerted any toxicity on OMM-1 cells or ARPE-19 cells after 24 h [91]. 

Overall, fucoidans exert few effects on the viability of ocular cells. However, purity might be of some importance. An overview of the fucoidans tested for their effects on the viability in ocular cells is given in Table 3.

An important task of RPE cells is the phagocytosis of shed photoreceptor outer segments in order to recycle used visual pigment and support regeneration of the photoreceptors [66]. A fucoidan from *Fucus vesiculosus* (from Sigma Aldrich, at 100 µg/mL) did not influence the phagocytic ability of primary RPE cells in short term stimulation. However, this fucoidan did reduce the wound-healing abilities of ARPE-19 and primary RPE cells [85]. Crude fucoidan from *Fucus distichus* subsp. *evanescens*, however, displayed a profound impact on phagocytosis of primary RPE, reducing it after 24 h (100 and 250 µg/mL), 3 d (1–250 µg/mL), and 7 d (100 and 250 µg/mL). This fucoidan also delayed the wound-healing of primary RPE cells [87]. These finding show the importance of testing the specific fucoidan to be used on the function of RPE cells. An overview of the effects of fucoidans on RPE function is given in Table 4.

## 3. Discussion

The treatment of age-related macular degeneration warrants new therapeutics that can be used at the early stages of the disease, in order to prevent disease progression and vision loss. Only recently were fucoidans suggested as possible therapeutics [40]. The rationale is tempting, as the possibility may exist to find a bioactive compound that might target various aspects of the disease at the same time. However, caution is needed, as the bioactivities of fucoidans differ profoundly, depending on various factors [60]. The studies summarized above document the first steps in the characterization of fucoidans as possible treatment options, which can act as a baseline and pave the way for further developments. Of course, there are limitations to these studies, inherent to all in vitro testing [67]. The effects of a fucoidan in cell culture may not reflect the effects of the fucoidan in the organism, as bioavailability cannot be modeled in cell culture. In addition, effects because of the interaction of cells in the tissue cannot be modeled either. Furthermore, a complex disease like AMD cannot be appropriately modeled in a dish, and testing certain stimuli can only be an approximation of the disease. Nevertheless, testing needs to start in the cell culture model before further studies in higher complex systems and animals can be considered.

Of the many species tested, *Saccharina latissima* and *Laminaria hyperborea* seem to be reasonable choices for further investigation and development, as fucoidans from both species decrease VEGF in ARPE-19 cells and RPE cells, and both may confer some protection against oxidative stress. Additionally, the studies indicate that high molecular weight may be preferable considering anti-VEGF function. Other factors that may be of importance are the degree of sulfation and the fucose content of the extract. Therefore, fucose-rich, high-molecular weight, and highly-sulfated fucoidans of the species *Saccharina latissima* and *Laminaria hyperborea* should be the fucoidans of choice for further development. Of great importance would be to test fucoidans in animal models to confirm the desired bioactivities in vivo. However, caution is warranted: Fucoidans tested for potential therapeutic use need to be thoroughly characterized and before any testing in animals can be considered, their potential has to be clearly proven in in vitro assays. Furthermore, the route of application has to be considered, as the bioavailability of fucoidans is still under investigation, looking at intravenous, topical, and oral applications [96,97,98]. While the bioavailability of orally-administered fucoidans has been considered rather low [99,100], absorption of fucoidans in the digestive tract and their presence in plasma and organs after oral application has been shown [96,98,101]. Moreover, orally-applied fucoidans have shown to be absorbed in humans [99,102]. The effects of orally-applied fucoidans on the retina, however, have not been evaluated so far and need to be studied in vivo. In addition, it has to be mentioned that animal models for AMD are actually very limited in their ability to model the human situation and usually only model some selected aspects [67]; e.g., mouse models of AMD hardly develop choroidal neovascularization [103]. In order to mimic CNV, laser-induced injuries are applied to the retina, which, however, induce angiogenesis as a self-limiting wound-healing reaction that is hardly comparable to a long-term chronic situation, as seen in AMD [67]. Therefore, great care has to be taken to select an appropriate model, and the use of different model systems is advised. As fucoidans seem to be suitable for the prevention of the progression, long term studies in chronic AMD models would likely be the most appropriate way to test their efficacy.

Oxidative stress and angiogenesis are two important (but not the only) pathogenic mechanisms of AMD. According to literature, fucoidans could also counteract lipid dysregulation [104] and inflammation [54,105]. It would therefore be of high interest to test fucoidans in these aspects, in order to find a fucoidan targeting multiple pathological mechanisms for further development. 

Fucoidans are natural products which are variable in structure and biological activities, depending on the origin, environmental influences, extraction methods, and the post-processing. In order to develop a product to be used in medical applications, a reproducible product of (in the best case) consistent chemical characteristics and high purity is needed. Furthermore, if such a fucoidan should prove useful as an AMD therapeutic, a sustainable source needs to be identified, to ensure that the use for man’s benefit does not result in the extinction of the benefactor. 

Taken together, fucoidans show exciting potential as a possible new treatment option for counteracting AMD progression, yet a lot of further research needs to be conducted in regard to bioactivity, availability, application, and in vivo efficacy. 

## 4. Materials and Methods 

In this review, publications concerning fucoidan in AMD-related research have been presented. Suitable publications were searched for in PubMed (National Library of Medicine), using the following search terms: fucoidan AND age-related macular degeneration (seven hits), fucoidan AND retinal pigment epithelium (seven hits); fucoidan AND ARPE19 (five hits); fucoidan AND retina (six hits); sulfated fucans AND age-related macular degeneration (seven hits); sulfated fucans AND retinal pigmented epithelium (seven hits); sulfated fucans AND ARPE-19 (five hits); sulfated fucans AND retina (six hits). Without doublings, a total of 10 publications were found and included in this study, of which seven are from our group. One of the papers was a hypothesis paper. A list of included studies can be found in Appendix A. 

## 5. Conclusions

Studies on fucoidan in in vitro assays have shown promising results concerning VEGF inhibition, and to a lesser degree, oxidative stress protection. Most promising species so far are *Saccharina latissima* and *Laminaria hyperborea*, preferably with fucoidans of high molecular weight and high fucose content. Further research is warranted to investigate additional beneficial effects, e.g., regarding inflammation. Pure, reproducible fucoidans need to be further developed and tested in in vivo studies. 

## Figures and Tables

**Table 1:** List of tested fucoidans, VEGF reduction.

Species	Origin	Conc [µg/mL]	Fucose [mol%]	TPC [μgGAE/mg]	MW [KDa]	ARPE-19	RPE
*Saccharina latissima*	North Atlantic	1–100	83.8	9.7	1407	Reduced at 10–100 µg/mL	Reduced at 10 µg/mL
*Fucus serratus*	Baltic Sea	1–100	40.6	50.3	605	Reduced at 1–100 µg/mL	No reduction
*Fucus vesiculosus*	Baltic Sea	1–100	59.2	35.1	1340	Reduced at 1, 50, 100 µg/mL	No reduction
*Fucus distichus* subsp. *evanescens*	Baltic Sea	1–100	96.1	25.8	188	Reduced at 1–100 µg/mL	No reduction
*Laminaria digitata*	North Atlantic	1–100	67.1	9.4	460	Reduced at 1–100 µg/mL	No reduction
*Laminaria hyperborea*	North Atlantic	1–100	97	n.d.	1548	Reduced at 50 and 100 µg/mL	Reduced at 50 µg/mL
*Laminaria hyperborea*	North Atlantic	1–100	97	n.d.	299	Reduced at 10–100 µg/mL	Reduced at 50 and 100 µg/mL
*Laminaria hyperborea*	North Atlantic	1–100	97	n.d.	26.9	Reduced at 50 and 100 µg/mL	No reduction
*Saccharina latissima*	North Atlantic	1–100	64.7	n.d.	>800	Reduced at 1–100 µg/mL	n.d.
*Laminaria digitata*	Baltic Sea	1–100	3.9	n.d.	322	Reduced at 10–100 µg/mL	n.d.
*Fucus vesiculosus*	Sigma Aldrich	1–100	n.d.	n.d.	n.d.	Reduced at 50 and 100 µg/mL	Reduced at 1–100 µg/mL
*Fucus distichus* subsp. *evanescens*	Baltic Sea	1–250	61.9	14.4	88.60	Reduced at 100 and 250 µg/mL	n.d.

References: [76,79,86,87,90,91]. Abbreviations: Conc = concentration; GAE = gallic acid equivalent; MW = molecular weight; n.d. = not determined; TPC = total phenol content; UM = uveal melanoma.

**Table 2:** List of tested fucoidans, oxidative stress protection.

Species	Origin	Conc [µg/mL]	Fucose [mol%]	TPC [µg GAE/mg]	MW [DDa]	OMM-1	ARPE-19
*Saccharina latissima*	North Atlantic	1–100	83.8	9.7	1407	Protective at 10–100 µg/mL	Protective at 10–100 µg/mL
*Fucus serratus*	Baltic Sea	1–100	40.6	50.3	605	Protective at 1–100 µg/mL	No protection
*Fucus vesiculosus*	Baltic Sea	1–100	59.2	35.1	1340	Protective at 1–100 mg/mL	No protection
*Fucus distichus* subsp. *evanescens*	Baltic Sea	1–100	96.1	25.8	188	Protective at 1–50 µg/mL	No protection
*Laminaria digitata*	North Atlantic	1–100	67.1	9.4	460	Protective at 1–100 µg/mL	No protection
*Laminaria hyperborea*	North Atlantic	1–100	97	n.d.	1548	No protection	Protective at 10 µg/mL
*Laminaria hyperborea*	North Atlantic	1–100	97	n.d.	299	No protection	No protection
*Laminaria hyperborea*	North Atlantic	1–100	97	n.d.	26.9	Protective	No protection
*Saccharina latissima*	North Atlantic	10–100	64.7	n.d.	>800	Protective at 1–100 µg/mL	No protection
*Laminaria digitata*	Baltic Sea	1–100	3.9	n.d.	322	No protection	No protection
*Fucus vesiculosus*	Sigma Aldrich	100	n.d.	n.d.	n.d.	Protective	n.d.
*Fucus distichus* subsp. *evanescens*	Baltic Sea	1–250	61.9	14.4	88.60	n.d.	No protection

References: [76,79,86,87,90,91]. Abbreviations: Conc = concentration; GAE = gallic acid equivalent; MW = molecular weight; n.d. = not determined; TPC = total phenol content; UM = uveal melanoma.

**Table 3:** List of tested fucoidans, viability.

Species	Origin	Conc [µg/mL]	Fucose [mol%]	TPC [µg GAE/mg]	MW [kDa]	OMM-1 Cell	ARPE-19
*Saccharina latissima*	North Atlantic	1–100	83.8	9.7	1407	Mixed	Increased (50 and 100 µg/mL)
*Fucus serratus*	Baltic Sea	1–100	40.6	50.3	605	Reduced (50 and 100 µg/mL)	Reduced (1–100 µg/mL)
*Fucus vesiculosus*	Baltic Sea	1–100	59.2	35.1	1340	Mixed	Reduced (50 µg/mL)
*Fucus distichus* subsp. *evanescens*	Baltic Sea	1–100	96.1	25.8	188	Mixed	No effect
*Laminaria digitata*	North Atlantic	1–100	67.1	9.4	460	Increased (10 and 50 µg/mL)	Increased (50 and 100 µg/mL)
*Laminaria hyperborea*	North Atlantic	1–100	97	n.d.	1548	No effect	Increased (50 µg/mL)
*Laminaria hyperborea*	North Atlantic	1–100	97	n.d.	299	Reduced (50 and 100 µg/mL)	No effect
*Laminaria hyperborea*	North Atlantic	1–100	97	n.d.	26.9	Reduced (10–100 µg/mL)	No effect
*Saccharina latissima*	North Atlantic	1–100	64.7	n.d.	>800	No effect	No effect
*Laminaria digitata*	Baltic Sea	1–100	3.9	n.d.	322	Increased (10–100 µg/mL)	Increased (100 µg/mL)
*Fucus vesiculosus*	Sigma Aldrich	100	n.d.	n.d.	n.d.	No effect	No effect
*Fucus distichus* subsp. *evanescens*	Baltic Sea	1–250	61.9	14.4	88.60	n.d.	No effect
*Dictyosiphon foeniculaceus*	Baltic Sea	1–100	38.7	11.0	194	Mixed	Increased (50 µg/mL)

References: [76,79,86,87,90,91,95]. Abbreviations: Conc = concentration; GAE = gallic acid equivalent; MW = molecular weight; n.d. = not determined; TPC = total phenol content; UM = uveal melanoma; Mixed: both increase and reduction were found.

**Table 4:** List of tested fucoidans, retinal pigment epithelium (RPE) function.

Species	Origin	Conc [µg/mL]	Fucose [mol%]	TPC [µg GAE/mg]	MW [KDa]	Phagocytosis	Wound Healing
*Fucus vesiculosus*	Sigma Aldrich	100	n.d.	n.d.	n.d.	No effect	Reduced
*Fucus distichus* subsp. *evanescens*	Baltic Sea	1–250	61.9	14.4	88.60	Reduced	Reduced

References: [85,87]. Abbreviations: Abbreviations: Conc = concentration; MW = molecular weight; n.d. = not determined; TPC = total phenol content.

## Abbreviations

AMD	Age-related macular degeneration
Conc	Concentration
GAE	Gallic acid equivalent
MDPI	Multidisciplinary Digital Publishing Institute
MW	Molecular weight
n.d.	Not determined
ROS	Radical oxygen species
RPE	Retinal pigment epithelium
TBHP	*Tert*-butyl hydroperoxide
TPC	Total phenolic content
UM	Uveal melanoma
VEGF	Vascular endothelial growth factor

## Appendix A

List of included studies: [40,63,76,80,84,85,87,90,91,95]. The following search terms were used: fucoidan AND age-related macular degeneration; fucoidan AND retinal pigment epithelium; fucoidan AND ARPE-19; fucoidan AND retina; sulfated fucans AND age-related macular degeneration; sulfated fucans AND retinal pigmented epithelium; sulfated fucans AND ARPE-19; sulfated fucans AND retina.
